# Applying a Primary Health Care Approach to Closing the Human Resource for Health Gaps for Immunization

**DOI:** 10.3390/healthcare12141449

**Published:** 2024-07-20

**Authors:** Folake Olayinka, Thomas S. O’Connell, Christopher Morgan, Maria Fernanda Monzon, Tokunbo Oshin, Tova Tampe, Alexandra Reed, Giorgio Cometto, Adolphus Trokon Clarke, Muhammad Ahmad Kazi, Jessica C. Shearer

**Affiliations:** 1 Public Health Institute, Via United States Agency for International Development’s Global Health Training, Advisory and Support Contract (GHTASC) Project, Washington, DC 20024, USA; folayinka@usaid.gov; 2 Department of Global and Environmental Health, New York University, New York, NY 10003, USA; tso2@nyu.edu; 3 Jhpiego, The Johns Hopkins University Affiliate, Baltimore, MD 21231, USA; christopher.morgan@jhpiego.org; 4 Ministry of Health, Corrientes W3400, Argentina; mfmonzon@gmail.com; 5 High Impact Countries, Country Programmes Delivery, Gavi, The Vaccine Alliance, 1218 Geneva, Switzerland; toshin@gavi.org; 6 Special Programme on Primary Health Care, World Health Organization, 1211 Geneva, Switzerland; tampet@who.int; 7 Health Workforce Department, World Health Organization, 1211 Geneva, Switzerland; comettog@who.int; 8 Ministry of Health, Expanded Program on Immunization, Monrovia 1000, Liberia; aclarke954@yahoo.com; 9 Federal Directorate of Immunization, Government of Pakistan, Islamabad 44000, Pakistan; drmakazi@gmail.com; 10 Independent Researcher, Arlington, VA 22207, USA; jess.shearer@gmail.com

**Keywords:** immunization, primary health care, human resources for health

## Abstract

This perspective is focused on the evidence on human resources for health (HRH) solutions for immunization, as a part of a primary health care (PHC) approach.. In the wake of the COVID-19 pandemic and 50 years since the Expanded Program on Immunization (EPI) clocks 50 years since its inception. was initiated, evidence and experience demonstrate the significant HRH gaps in many countries and globally, and how countries are seeking innovative ways of closing them with limited resources. The aim of this perspective article is to highlight the growing gap between the needs and the realities related to health workforce for PHC, including immunization, and to call for increasing the visibility of HRH within global and national immunization agendas. This perspective highlights key guidelines and tools to improve HRH, such as integrating immunization and primary health care, addressing the mental health needs of the health workforce, addressing gender-related issues, rationalizing the roles and composition of PHC workforce teams, and meeting the surge requirements related to health emergencies.

## 1. Introduction

Health workers have been the backbone of immunization programs for the Expanded Program on Immunization’s (EPI’s) 50 years. The World Health Organization (WHO) established the EPI in 1974 to ensure all children have access to live-saving vaccines. During the last 50 years of the EPI, the number of children vaccinated annually has increased dramatically, from 17% of the world’s children in 1980 to 85% of children in 2022 [1]. The number of vaccines administered by health workers has increased from 6 in 1974 to 13 today [2], with other new vaccines becoming available across the life course (In 1974, EPI scheduled Bacillus Calmette–Guérin (BCG), diphtheria, pertussis, tetanus, polio, and measles. In 2024, the standard WHO schedule includes these vaccines, plus Haemophilus influenzae type B (Hib), Hepatitis B (HepB), rubella, pneumococcal disease (PNC), rotavirus (Rota), human papillomavirus (HPV), and COVID-19 (for adults) vaccines) [3]. The health workforce who deliver these services has not grown at the same rate, and yet we depend on them to provide high-quality vaccination services alongside other essential primary care services, beginning with patient education through to service delivery and monitoring.

The Immunization Agenda 2030 (IA2030) highlights a vision for ensuring global equity in immunization, with no one left behind, through a strong emphasis on primary health care (PHC) [4]. Achieving IA2030 will require well-motivated human resources for health (HRH) in the right numbers, equitably distributed, and with the required competencies and system support to deliver quality services. The same is true for achieving the Sustainable Development Goals (SDGs) and the Universal Health Coverage (UHC) targets [5]. IA2030 Strategic Priority 1 (“Immunization Program for PHC and UHC”) aligns these global and national goals through its emphasis on strengthening immunization programs as part of PHC, thereby contributing to UHC, and by monitoring objectives related to (1) ensuring the availability of an adequate, effective, and sustainable health workforce; and (2) reinforcing and sustaining strong leadership, management, and the coordination of immunization programs at all levels [4]. The IA2030 Framework for Action: Immunization for Primary Health Care [6] builds on the WHO-UNICEF Operational Framework for PHC, and lays out practical actions that countries can take, using a PHC approach aligned to the four strategic and ten operational PHC levers [7]. The aim of this perspective article is to highlight the growing gap between the needs and the realities related to the health workforce for PHC, including immunization, and to call for increasing the visibility of HRH within the global and national immunization agendas.

## 2. The Issue: A Widening Gap between Available Health Workforce and Growing Needs

The relationship between health worker density and UHC has been well documented [8], but recent data suggest the shortage of health workers is not being addressed quickly nor comprehensively enough to achieve IA2030, SDG, and UHC goals [9]. These shortages were further exacerbated by the COVID-19 pandemic [10,11]. In 2020, the global health workforce shortage was estimated to be 15.4 million health workers, including 7.07 million nursing personnel, 2.66 million medical doctors, 410,000 midwifery personnel, 290,000 pharmacists, 260,000 dentists, and 4.69 million categorized as other occupations [12].

Within these estimates, huge inequities exist between and within regions and countries, with high-income countries having a health worker density that is 6.5 times that of low-income countries [12]. The composition and distribution of the health workforce varies across countries and are dependent on several factors, such as the overarching health workforce policies and strategies, the disease burden, and the will and capacity of governments to invest in health care education and employment. In 2023, the WHO identified 55 countries as “vulnerable” regarding the availability of health workers required to achieve the UN Sustainable Development Goal target for universal health coverage (UHC) by 2030 [13]. Thirty-seven of these countries are in the Africa region, followed by eight in the Western Pacific, six in the Eastern Mediterranean and three in the South East Asia regions. Many countries on this list also bear the greatest burden of vaccine-preventable diseases.

There are several challenges that affect health workers’ availability, including inadequate HRH planning, disparities between education strategies and employment opportunities, and poor remuneration. But, particularly in low- and middle-income countries, there is inadequate budget space for the health workers required to meet population health needs [14]. Issues with distribution also exist, especially where there is a rural–urban maldistribution caused by inadequate incentives to work in rural and hard-to-reach areas. We need to know more about what motivates health workers supporting PHC -including immunization -and what is required to enable them to sustain and grow careers in health, ensure decent work environments exist, and address gender inequities that hamper advancement.

There is growing insight into the availability, feasibility, and functions of community health workers (CHWs), and WHO advises its member states to progressively integrate CHWs within the formal health system and remunerate them as part of an integrated health workforce approach [15,16]. The community health workforce is an important but underutilized resource in the provision of basic promotive and preventive primary care services, especially in hard to reach areas. While the levels of education and remuneration of CHWs range widely between countries, they are not trained to the level of nurses or doctors. For immunization, CHWs in many countries are trained to mobilize families for immunization, provide information and education, and trace defaulters [17,18,19]. A recent review of CHW programs found that 20 countries have used CHWs to administer vaccines, and that well-trained and supervised CHWs can effectively expand the immunization health workforce under the right conditions [19]. This more ambitious use of CHWs should be part of longer-term strategies that begin with efforts to formalize the roles, the certification and motivation of CHWs, and which ensure the most efficient and safest allocation of tasks across the health workforce.

There is a growing body of literature on the required competencies of the immunization health workforce [20], strategies to improve both EPI skills and capabilities [21], and standard training materials for health workers and managers [22,23]. However, HRH is an issue within immunization that exemplifies the need for broader PHC and intersectoral approaches. For example, an orientation towards team-based models of integrated service delivery using a PHC approach can improve resource use, decrease missed opportunities for vaccination, and enhance patient outcomes. The design and implementation of these models requires strong national and, in decentralized contexts, sub-national institutions to develop standards, ensure quality and regulate implementation; all of these are related to the ongoing need to increase financial resources for health [14].

## 3. Regional Perspectives

An assessment of the health workforce stock in 2020 projects the Africa region to have a 5.3 million shortfall of health workers by 2030 [24], representing over half of the world’s projected shortage of health workers of 10.2 million in 2030. While the projected health worker shortage in 2030 represents some improvement from the 2020 estimate of 5.7 million, the shortage was only 5.1 million in 2013 and made up only one quarter of the global shortage [12]. Member states and regional partners are collaborating to address the health workforce gaps in the region through the development of the African Health Workforce Investment Charter. The Charter is focused on the alignment and catalytic investments to reduce the inequities in the health workforce, particularly in the 36 countries with the most critical gaps. Other instruments to potentially address the gap include the African Union Commission’s COVID-19 Recovery Framework for Africa and Africa Centres for Disease Control and Prevention’s New Public Health Order. These high-level pathways need to be implemented consistently to be effective to fill the gaps, by sustainably increasing public sector and development partners’ investments into education, jobs and decent working conditions for health workers.

The 2019 *Monitoring progress towards the vision of Health Islands in the Pacific* report found that only half of the Pacific island countries reporting on skilled health worker density met the benchmark of 4.4 per 1000 [25]. Acknowledging health workforce shortages, a recent study highlighted the optimization of the composition of PHC teams with the most rational and cost-effective distribution of roles and responsibilities, particularly at the community level, and underscored the opportunities for strengthening the PHC workforce [26]. Another study points to the challenges of high rates of staff turnover, which undermines efforts to reach adequate health workforce levels [27]. At the leadership level, the 14th Pacific Community Pacific Heads of Health Annual Meeting delved into the issue of the health workforce pipeline, retention, and integrated primary care service delivery models as major strategic approaches to achieve UHC [28].

## 4. Gendered Dimensions of the Health and Social Care Workforce

Women currently make up 67 percent of the health and social sector workforce worldwide [29]. Women are estimated to provide essential health services for five billion people worldwide. The financial value of women’s input into health systems is estimated to be over USD 3 trillion annually [30]. Yet, compared to men, women’s contributions to health and the health labor market remain markedly undervalued [31]. They regularly face challenges impacting their job performance, compensation, and career advancement [32]. These challenges include gender discrimination, resulting in unequal pay and treatment in the workplace, inadequate workplace conditions that safeguard their health and security, or advancement opportunities; violence and sexual harassment; restricted independence or mobility outside the home; and the burden of balancing pregnancy and family expectations with their job [33]. An analysis of the gender pay gap in 54 countries shows that women’s mean hourly wage is overwhelming less than that of men in the health and care sector. The average regional gender pay gap is the highest (about 25%) in the Africa region, and the lowest in the Western Pacific and South East Asia regions (5–10%) [29]. Additionally, the burden of unpaid health and social care work commonly carried out by women and girls is largely unacknowledged [34]. Equity issues pertaining to decent work free from all forms of discrimination; harassment, including sexual harassment; the gender pay gap; and occupational segregation by gender and leadership are important for all member states to address the protection and retention of existing health and care workers, if the 15.4 million health worker shortfall is to be redressed in an equitable, inclusive and sustainable way [35,36]. Gender equality is key to building resilient health systems, and gender transformative health and social care policies are key to achieving gender equality globally.

## 5. Lessons Learned from COVID-19

The COVID-19 pandemic underscored the critical role of HRH to not only respond to the COVID-19 pandemic, but also address recovery needs and maintain access to other essential health services, including immunization. As noted by Bourgeault et al., “It seems to have taken this pandemic for all of us to explicitly value health workers” [37]. The International Council of Nurses reported the crucial role of nurses in building awareness and trust of COVID-19 vaccines as a determinant of vaccine coverage [38]. The COVID-19 pandemic also offered an opportunity to implement new HRH strategies to optimize health worker skills and meet surge requirements, such as task sharing and task shifting across cadres, reduced pre-service training or changes in licensing requirements, the use of retired health workforce, and the expanded use of volunteers. Countries also adapted service delivery strategies, such as telemedicine and scheduling appointments, which improved access to health workforce while improving health workers’ working conditions and efficiency [39]. These actions likely helped maintain access to essential health services, but still it is estimated that 67 million children either partially or fully missed vaccines during this period [40]. Meanwhile, not all of the short-term innovations in the health workforce have been sustained after the emergency pandemic funding was no longer available.

The COVID-19 pandemic exacerbated health workers’ stress and work overload, with 50 percent of health workers experiencing burnout from the burden of extra work and/or negative community sentiment towards some public health responses [41]. Other issues included those related to discrimination, lack of insurance, and access to sufficient personal protective equipment, especially in the early stages of the pandemic [38]. The COVID-19 pandemic strained existing HRH, including those providing immunization services and other cadres, such as surveillance officers. These service providers were also deployed to emergency services for the COVID-19 response, which impacted the capacity to continue to provide routine essential immunizations. The rebound of immunization programs is closely linked with the availability of well-performing and an adequately distributed health workforce, among other factors, such as vaccine confidence.

## 6. Case Studies: Approaches to Leveraging and Strengthening the PHC Workforce

The following case studies share good HRH practices across a range of countries, which immunization and PHC policymakers should consider to improve health workforce availability, competencies, and satisfaction.

*Leveraging a trusted community workforce in Liberia:* Liberia has relied heavily on its Community Health Workers (CHWs) to restore basic services across the country, especially through its flagship National Community Health Assistant Program. CHWs play an important role in building the trust of communities in health services, and showed that particularly in the response to Ebola. During the COVID-19 pandemic, the National Community Health Assistants led efforts to educate their communities on disease transmission, handwashing, and home isolation protocols. They also supported efforts to screen and refer household members in their communities. Beyond COVID-19, the flagship program has seen over 4000 community health workers undertake more than five million home visits to refer for vaccination, treating nearly one million cases of childhood pneumonia, malaria, and diarrhea [42,43].

*Long-term institutionalization of team-based PHC in Brazil:* Since the late 1980s and into the 2000s, Brazil has implemented multiple programs to build capacity in HRH training and management, and to close the gap between HRH availability and need for primary care [44]. The Family Health Strategy in Brazil—which includes a team-based model of care integrating multiple cadres of health workforce—is the main approach that enables access to health care for around two-thirds of the population [45]. The institutionalization of CHWs into family health teams took over three decades to achieve, but they are now part of these teams, which also include a nurse, a nurse assistant, and a physician. Family health teams’ tasks include community mobilization, data collection and management, health prevention and promotion, planning, vector control, social care, and environment-related activities [46]. This program embodies a holistic approach to enhance the health of vulnerable communities, and has helped reduce the mortality of children under five years of age by 75% and maternal mortality by almost 60% [47]. Between 1990 and 2009, Brazil increased nurses by 500% and physicians by 66% [44].

*Leveraging short- and long-term recruitment strategies in Pakistan to fill HRH gaps:* In Pakistan, federal and provincial government health authorities are committed to the PHC model. Many health sector challenges in Pakistan are related to HRH: a severe shortage of health workforce, especially nurses, midwives, and lady health workers; the imbalanced geographical distribution of the health workforce, including between urban and rural areas; imbalanced skill combinations; inadequate skills; poor job satisfaction and work environment; and out-migration [48]. For a country with one of the lowest health worker densities in its region, the health worker surge required for COVID-19 immunization severely tested this aspiration, as health professionals not well-versed in immunization required rapid skill training to staff mass-vaccination centers, and PHC teams needed communications support to respond to misinformation. To ensure a smooth rollout of COVID-19 vaccines and address the shortage of vaccinators, Gavi’s COVID-19 vaccine delivery support (CDS) grants enabled the hiring of 2667 vaccinators, and the country hired an additional 1889 vaccinators at the PHC level (Gavi’s COVID-19 vaccine delivery support is a funding package contributed through its multilateral partnership to support the roll-out and scale-up of COVID-19 vaccines). This was in addition to a hiring surge of short-term contract health professionals, COVID-19 intensive care units and field hospitals. As emergency responses are wound down, it is of even greater importance to act on the HRH strategy to invest in meeting workforce gaps to deliver strong primary, preventive, and promotive health care. Pakistan is already making efforts to invest in meeting workforce gaps, such as for the lady health workers’ workforce to be increased from 89,282 in 2021 to 135,230 by 2027–2028, as well as for them to be trained on immunization and primary care [49]. Recent initiatives, such as the Public Private Partnerships and the Civil Society Organizations engagements, have substantiated the health service delivery and demand generation for the achievement of the national goals of PHC.

## 7. Actions

To achieve IA2030, UHC and the SDGs, countries must reorient their health systems towards PHC, which includes reimagining the health workforce. Actions to address immediate needs include mapping out and coordinating across sectors to fill the most critical gaps; developing surge plans for emergencies; and ensuring adequate protection, safety and incentives for the health workforce. The IA2030 Framework for Action: Immunization for Primary Health Care discusses how to strengthen human resources as follows: advocate for policies to attract and retain a fit-for-purpose and motivated health workforce at the primary care level, especially in remote settings; integrate priority PHC topics into immunization training and vice versa; ensure regular integrated supportive supervision visits for PHC and immunization; optimize digital tools for learning and performance; develop standard operating procedures, training materials, and job-aids for continuous learning through hybrid and blended approaches; and the delivery of high-quality integrated services [6].

The mental well-being of health workers warrants careful attention [50]. First, by ensuring that workloads are appropriate, and second, by providing emotional support to staff, including access to services and resources to identify and manage work-related stress, depression, anxiety, and trauma [41]. Much more attention needs to go towards improving working conditions and the remuneration of the health workforce.

While gender discrimination and inequality are globally acknowledged, regional and country progress vary in terms of addressing it, including upholding rights and institutionalizing protections against all forms of gender discrimination and inequality [51]. Even in countries with relatively similar vaccination rates, female children may be vaccinated later than males, increasing morbidity and mortality from vaccine-related illnesses [52]. Global and regional health partners need to not only ensure the existence of regional and national pro-equity policies, but also ensure the legal backing and frameworks that guarantee protections are actualized in each region [53].

Health workforce productivity may be improved through rationalizing the roles and composition of primary health care teams; for example, through team-based care models, and through workforce redeployment [54]. Such changes require careful consideration of the various tasks involved in supporting and delivering vaccination, and taking a new look at who is best placed to support these. Revising skill combinations and role delegation must be carefully planned to ensure the health workers are educated, authorized to deliver the services for which they are trained, supervised, supported and have the tools and competency to perform new roles, and are not overburdened [55,56].

Adequate human resources are essential for strengthening essential public health functions, especially when considering health workers’ role in preventing and responding to emergencies [57]. This can include adapting to virtual care and telehealth during emergencies, when reducing face-to-face interactions is needed. Providing health workers with training on the use of technologies, including data protection, as well as supporting access to virtual care among patients, should be considered.

Prioritizing closing the gaps in the Africa region must be a top priority, as more than half of the world’s HRH shortages are projected to be occurring there. Sharing the best practices on reducing the gender pay gap from countries in the Western Pacific and South East Asia regions will be helpful for cross-regional learning.

Long-term approaches and planning are required to ensure sustained gains and adjustments as health needs change and health workers retire. These include strategic planning and budgeting for the health workforce within the context of stronger PHC, and allocating adequate resources for systems strengthening through a PHC approach, including in the context of health emergencies [14]. The COVID-19 pandemic has provided important learnings to optimize health worker skills, meet surge requirements, and adapt service delivery strategies. Strengthening the health workforce not only improves the effectiveness of current health systems but also improves the ability to prevent, prepare for, and respond to future pandemics. Countries should leverage an intersectoral approach that includes finance, labor, public service, private sector, and education, and continue to build a PHC-oriented health workforce that can deliver basic preventive, promotive, and curative services to their neighbors and communities.

## 8. Conclusions

In line with the WHO Global Strategy on Human Resources for Health, and recent urgent calls for all countries to invest in their health workforces, a renewed emphasis on investment in education and jobs for health workers should become a top priority as part of reorienting health systems towards PHC [9,14]. Achieving IA2030 and UHC goals through PHC will remain elusive if gaps in human resources are not addressed, particularly in recruiting, supporting and retaining a fit-for-purpose health workforce.

An adequately skilled, appropriately distributed, and motivated health workforce is essential to achieve immunization for all, as well as foundational to strengthening the PHC-oriented health systems that achieve UHC and the health-related SDGs. This requires political and country leaders, multilateral institutions, development partners, public and private sector stakeholders, civil society, and community leaders to take concerted, proactive, inclusive and collective action to make this a reality. Low-income contexts must not be left behind. One of the biggest lessons from COVID-19 is that we must commit to solidarity and global safeguards in optimizing the health workforce around the globe to achieve health and development goals, and certainly to prevent and respond to future pandemics.
